# MSI-FusionNet: a multi-modal spectral-image fusion network for sorghum variety identification

**DOI:** 10.1016/j.fochx.2025.103137

**Published:** 2025-10-09

**Authors:** Xinjun Hu, Mingkui Dai, Anjun Li, Ying Liang, Wei Lu, Jiahao Zeng, Jianheng Peng, Jianping Tian, Manjiao Chen, Liangliang Xie

**Affiliations:** a School of Mechanical Engineering, Sichuan University of Science and Engineering, Yibin 644000, China; b Anhui Province Key Laboratory of Intelligent Solid-state Fermentation Technology, Bozhou 236800, China; c Anhui GujingGongjiu Co., Ltd, Bozhou 236800, China

**Keywords:** Sorghum, MSI-FusionNet, Multi-modal data fusion, Variety identification

## Abstract

Compositional differences among sorghum varieties influence the brewing process, flavor characteristics, and overall quality of Baijiu. This study proposes a Multi-Modal Spectral-Image Fusion Network (MSI-FusionNet) data fusion model for rapid and accurate identification of sorghum varieties. This model integrates one-dimensional spectral data obtained through hyperspectral imaging with two-dimensional image data captured using industrial microscopes. The model identifies 12 sorghum varieties with an accuracy of 93.33 %. Compared with using spectral or image data alone, MSI-FusionNet improves accuracy by 11.11 % and 29.63 %, respectively. To balance performance and efficiency, various classic 2D convolutional neural network (2DCNN) architectures were evaluated. The MSI-FusionNet model with ShuffleNetV2 as the 2DCNN structure demonstrated superior efficiency, significantly reducing model complexity and computational cost while maintaining high accuracy. MSI-FusionNet offers an efficient and accurate solution for identifying sorghum varieties for liquor enterprises, supporting the stability of Baijiu flavor and quality, and providing valuable technical support for the brewing industry.

## Highlights

• Developed MSI-FusionNet, a multi-modal fusion network integrating spectral and microscopic image data.
• Achieved 93.61 % accuracy for classifying 12 sorghum varieties using multi-modal fusion.
• Improved classification accuracy by 10.7 % over spectral data and 29.91 % over image data alone.
• Incorporated ShuffleNetV2 to reduce model complexity while maintaining high performance.

## Graphical abstract

**Figure:**
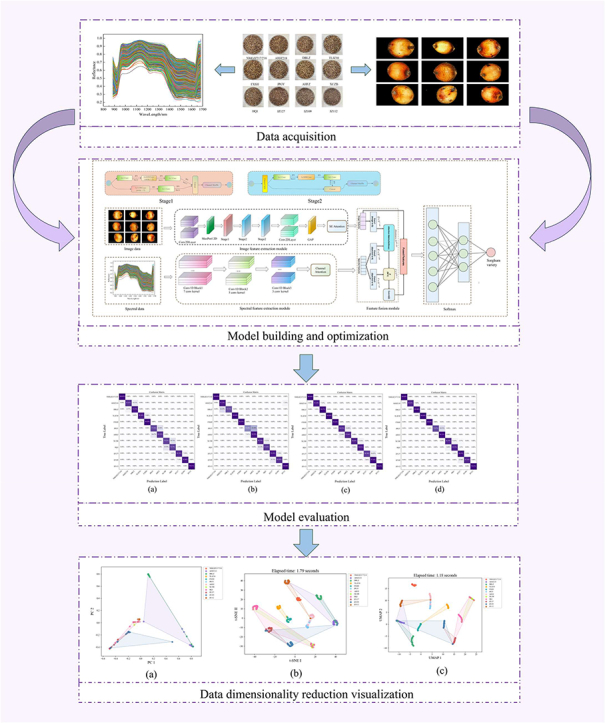

## 1 Introduction

Sorghum is a key raw material in Baijiu production, and variations in composition can significantly impact the brewing process, flavor, and quality of the final product (Huang et al., 2022). As such, accurately identifying and selecting high-quality sorghum varieties is crucial for producing premium Baijiu. Traditional identification methods primarily rely on manual assessment, morphological characteristics, or chemical analysis. Manual approaches and morphological traits suffer from subjectivity, low efficiency, and susceptibility to environmental conditions (Han et al., 2024). While chemical methods offer high accuracy, they involve complex sample preparation, are time-consuming, and costly (Fei et al., 2023). These limitations make them unsuitable for the modern Baijiu industry, which demands fast, accurate, and non-destructive identification techniques. Hyperspectral imaging (HSI) is a non-destructive technique that captures both spectral and spatial information simultaneously (Hu et al., 2024). Compared with traditional methods, HSI offers rapid, accurate, and contactless detection, and has been widely used in crop variety identification—such as wheat (Jiang et al., 2023; Zhao et al., 2022), corn (Wang & Song, 2023; Zhang et al., 2024), and rice (Edris et al., 2024; Ge et al., 2024).

In recent years, deep learning (DL)—particularly convolutional neural networks (CNNs)—has gained prominence (Rong et al., 2020) due to their ability to autonomously learn from raw data and automatically extract key features, effectively overcoming the limitations of traditional feature engineering (Ortac & Ozcan, 2021). DL has demonstrated strong potential in HSI-based classification tasks. Zhao et al. combined principal component analysis (PCA) with a spectral-image CNN to identify 6 sorghum varieties with 98.64 % accuracy (Zhao et al., 2024). Nie et al. used a deep CNN to classify up to 6 hybrid okra and loofah seed varieties, achieving classification accuracies above 95 % (Nie et al., 2019). Zhu et al. applied a one-dimensional (1D)CNN with channel attention and transformer modules to classify 15 flaxseed varieties, achieving 95.26 % accuracy (Zhu et al., 2025). Additionally, Meng et al. used a hybrid CNN to identify 14 rice varieties with 98.93 % accuracy (Meng et al., 2022). Zhang et al. employed a dual attention feature fusion network to identify 10 soybean varieties, achieving 96.15 % accuracy (Zhang et al., 2025). These results highlight the excellent performance of DL technology, especially CNNs, in crop variety classification using hyperspectral data. However, despite the significant advantages of HSI, its relatively low spatial resolution limits the accurate identification of fine-grained features in sorghum seeds (Hu, Dai, Peng, et al., 2025). To address this, this study incorporates high-resolution images captured by industrial microscopes, which provide structural details of the sorghum surface, such as texture, color, and morphology. By combining 1D spectral data from HSI with two-dimensional (2D) image data captured using industrial microscopes, we developed MSI-FusionNet—a multi-modal network that uses both 1D and 2D convolutions. The model extracts and fuses spectral and image features to accurately classify 12 sorghum varieties. To further optimize model performance, we compared several classic 2DCNN architectures and used dimensionality reduction to visualize feature separability and classification results.

In recent years, low-rank representation methods have achieved remarkable progress in hyperspectral image reconstruction and feature extraction. The reconstruction method based on deep low-rank tensor representation (Dian et al., 2025b) effectively improved spectral reconstruction quality while reducing the parameter scale by introducing a tensor decomposition mechanism. Subsequently, the low-rank Transformer framework (Liu et al., 2024) further incorporated low-rank modeling concepts into spatial–spectral fusion tasks, significantly alleviating computational overhead while modeling global dependencies. Meanwhile, various innovative strategies have been proposed for hyperspectral and multispectral fusion. For example, the zero-shot super-resolution method (Dian et al., 2023) quantitatively estimates the spatial and spectral responses of imaging sensors to construct training pairs directly from test data, thereby achieving efficient hyperspectral sharpening without external data. Another category, optimization-based low-rank regularization methods (Dian et al., 2025a), introduced a generalized tensor nuclear norm that extends tensor nuclear norm regularization from the third mode to arbitrary modes, effectively modeling spatial–spectral correlations and overcoming mode permutation sensitivity, thus achieving superior performance in terms of fusion accuracy and robustness. These studies on low-rank modeling, tensor regularization, and self-supervised sharpening provide important references for the multimodal fusion architecture design proposed in this paper. The main contributions of this study are as follows:

(1) A multimodal dataset comprising twelve sorghum varieties was constructed, including both hyperspectral and microscopic image data;

(2) A lightweight multimodal fusion network, MSI-FusionNet, was designed, which employs a cross-modal attention mechanism to achieve effective integration of heterogeneous features;

(3) For the image modality, several commonly used two-dimensional CNN backbones were compared to balance recognition accuracy and computational efficiency, and ShuffleNetV2 was ultimately selected to achieve lightweight implementation;

(4) In the sorghum variety identification task, MSI-FusionNet achieved an accuracy of 93.33 %, significantly outperforming single-modality approaches.

## 2 Materials and methods

### 2.1 Sample preparation

To comprehensively analyze the impact of different geographical origins on the quality of brewing sorghum, we selected 12 high-quality brewing sorghum varieties from China's major sorghum-producing regions, including Inner Mongolia Jiza 217/218 (NMGJZ217/218), Aohanqi Jiza 218 (AHJZ218), Tongliao Jiza 30 (TLJZ30), Aohanqi Fangze (AHFZ), and Xincheng Zhongbang (XCZB); Northeast Liaoza (DBLZ), Fuxin Songhang (FXSH), Jianping Guyuan (JPGY), and Hanqing No.1 (HQ1); as well as Jiza 127 (JZ127), Jinza 109 (JZ109), and Jinza 112 (JZ112). To ensure sample representativeness and data reliability, 300 plump and intact seeds were selected from each variety, totaling 3600 seeds from the 12 varieties, for subsequent experimental analyses.

### 2.2 Hyperspectral instruments and data acquisition

This study employed an HSI system that was independently developed by Jiangsu Shuangli Hepu Technology Co., Ltd. The system included a hyperspectral camera, a support frame, a whiteboard, data acquisition software, and four halogen lamps. The spectral acquisition range of the hyperspectral camera was 886–1735.34 nm, with a spectral resolution of 5 nm. The acquired HSI were three-dimensional cube data containing 512 bands. To ensure acquisition stability, the camera was preheated for 15 min. Software parameters were adjusted to set the exposure time at 2 ms, forward speed at 0.075 cm/s, backward speed at 0.2 cm/s, and the vertical distance between the sample and camera at 40 cm. The center wavelengths for R, G, and B channels were set at 954.15, 927.55, and 904.28 nm, respectively. To obtain high-quality hyperspectral image data, sorghum grains were neatly arranged in a 5 × 5 matrix, ensuring that each sample could be completely captured. Meanwhile, to eliminate the effects of camera dark current and uneven halogen light source illumination on the hyperspectral image data quality and to improve data reliability and accuracy, black-and-white correction processing was performed on the acquired HSI data (Bai et al., 2020).

### 2.3 Spectral data extraction

**Fig. 1:**
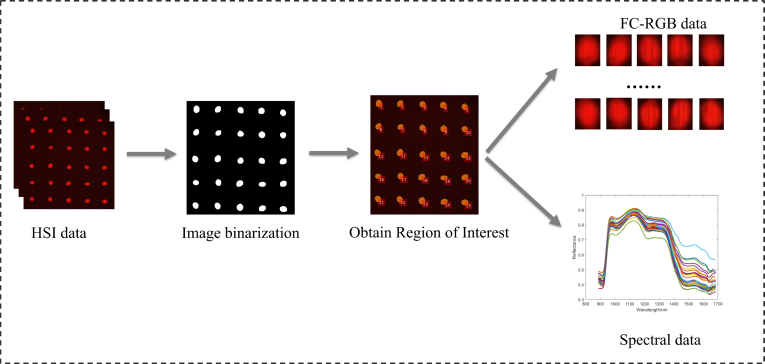
Extraction of the spectral data and FC-RGB image data.

To accurately extract the spectral data of sorghum grains, this study developed an automated ROI extraction workflow on the corrected hyperspectral images. First, background regions were removed through binary preprocessing (threshold 70/250) and polygon masking to obtain a rough effective range. Subsequently, the Otsu threshold segmentation algorithm (threshold 25/250) was applied for fine segmentation, combined with morphological operations to remove isolated noise and fill small holes within grain regions, making grain contours more complete. Next, all regions were preliminarily identified using connected component labeling, and noise regions that were too small were filtered out through an area threshold (80 pixels), retaining only complete grains. The retained regions were renumbered and spatially sorted based on centroid coordinates (first divided into 5 rows along the Y-axis, then sorted from left to right along the X-axis within each row), thereby obtaining 25 independent regions of interest (ROIs) in each sample (as shown in Fig. 1). On this basis, the spectral reflectance of all pixels within each ROI was extracted and averaged to serve as the one-dimensional spectral data for that grain. Meanwhile, corresponding pseudocolor images (FC-RGB) were also retained for subsequent visualization and verification. During the ROI definition process, background and external impurities were excluded, while small amounts of endogenous particles attached to grain surfaces and common surface defects (such as black spots, cracks, etc.) were retained to ensure the authenticity and representativeness of the spectral data.

### 2.4 Industrial microscope and image acquisition

**Fig. 2:**
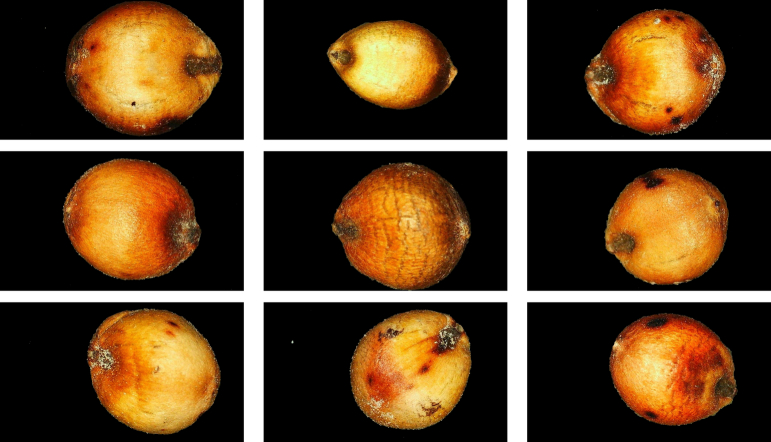
Industrial microscope images of sorghum grains.

Two-dimensional images of sorghum were acquired using an industrial microscope equipped with a 7-in. high-definition LCD (G1200). The industrial microscope used in this study comprised three core components: an optical and imaging system, a display and control system, and a mechanical structure. The digital magnification can reach up to 1200 times, with an imaging resolution of 720P (1280 × 720), ensuring high-image clarity. During the image-acquisition process, sorghum grains were placed on the microscope stage, and the focus was manually adjusted to ensure that the sorghum images were presented completely and clearly. To accurately analyze the image features of sorghum grains, the GrabCut algorithm was used to segment the background of the sorghum images and extract the foreground regions of the sorghum grains. The extracted foreground regions were then seamlessly merged with a pure black background. This processing method effectively eliminated background interference and highlighted the image features of sorghum grains, such as the shape, size, texture, and color. Fig. 2 displays the processed industrial microscope images (IMI) of sorghum grains.

### 2.5 Construction of multimodal spectral-image fusion network (MSI-FusionNet)

**Fig. 3:**
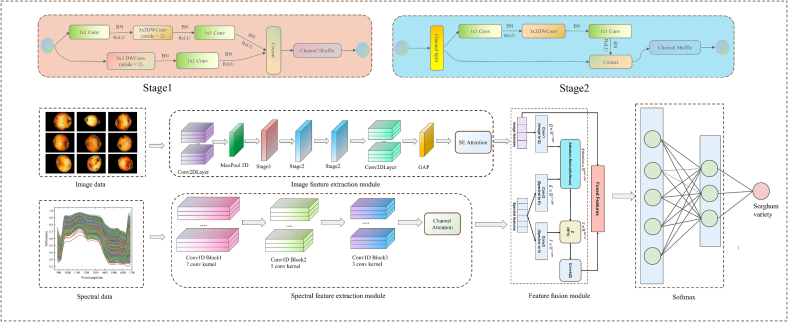
Multimodal Spectral-Image Fusion Network.

CNNs have been widely applied in multiple fields owing to their powerful feature extraction and representation learning capabilities, achieving remarkable outcomes (Do et al., 2025; Hou et al., 2025; Ren et al., 2025). The widespread attention to CNNs can primarily be attributed to their ability to automatically learn and extract the key features from raw data, thereby overcoming the limitations of CNNs that rely on manually designed features (Hu, Dai, Li, et al., 2025). This advantage enables CNNs to demonstrate performance superior to the conventional methods when processing data types, such as images and sequences. Based on the dimensionality of the convolution operation, CNNs can be categorized into three types: one-dimensional convolution (1D), two-dimensional convolution (2D), and three-dimensional convolution (3D). 1DCNNs are mainly used for processing sequence data, 2DCNNs are most widely applied in image processing tasks, while 3DCNNs are suitable for data with spatiotemporal characteristics such as videos. In this study, based on the characteristics of the sorghum data used, we constructed an MSI-FusionNet based on 1DCNN and 2DCNN, with its model structure shown in Fig. 3. Considering that the sorghum data includes one-dimensional spectral data and two-dimensional image data, 1D convolutions and 2D convolutions were adopted as feature extractors for spectral data and image data, respectively. Through convolution operations, the network can automatically learn and extract key features from each modal data. After feature extraction, the spectral features and image features are input into the cross-modal feature fusion module to achieve multi-modal information fusion. The fused features are then processed through fully connected layers and finally normalized by softmax to obtain the category probability output. The proposed MSI-FusionNet integrates multiple advanced deep learning techniques to fully leverage the complementary advantages of hyperspectral data and high-resolution images. The spectral branch employs a 1D-CNN structure, effectively capturing local spectral correlations and subtle absorption features along the wavelength dimension. The image branch utilizes ShuffleNetV2, a lightweight network that balances accuracy and computational efficiency, extracting rich spatial and texture information from high-resolution microscopic images while maintaining a compact model size suitable for industrial deployment. The model adopts a mid-level fusion (feature-level fusion) strategy, achieving deep interaction of high-level features from both modalities through cross-modal feature fusion before the classification layer. This approach preserves the independent expressive power of each modality while enhancing feature complementarity. The end-to-end network structure allows the feature extraction processes of both branches to be directly guided by the classification task, resulting in more optimized feature representations. Compared with traditional handcrafted feature fusion or shallow learning methods, MSI-FusionNet demonstrates higher accuracy and robustness in sorghum variety recognition.

#### 2.5.1 Spectral feature extraction module

To fully utilize the spectral data of 480 effective wavelengths, we constructed a one-dimensional convolution module. This module consists of three cascaded convolution blocks, each containing two convolutional layers. The kernel sizes in the three convolution blocks are 7, 5, and 3, respectively, all with a stride of 2, using zero padding of size 1 to maintain the spatial dimensions of the feature maps. By applying convolution kernels of different scales, this module can extract features of different scales from the spectral data. To optimize the training effect of the model, the Rectified Linear Unit (ReLU) activation function and batch normalization technique were applied after each convolutional layer. The ReLU activation function introduces a non-linear transformation, enabling the model to learn and extract complex feature representations while effectively alleviating the vanishing gradient problem (Clevert et al., 2015). By normalizing the outputs of the convolutional layers, the training process of the model was accelerated and its performance was enhanced (Ioffe, 2015). The detailed parameters of the 1D convolutional structure are summarized in Table 1. After convolutional feature extraction, the spectral data was output as a 128-dimensional feature vector through the fully connected layer L1, which was then passed to the shared fully connected layer S1, and finally compressed into a 64-dimensional spectral feature representation. To further improve the model's performance, a channel attention mechanism was introduced into the spectral feature-extraction process, which adaptively learns channel weights to emphasize information-rich channels and suppress the less important ones (Yang et al., 2024). Specifically, first, global average pooling was performed on the input feature map to obtain a channel descriptor that represents global information of each channel; then, non-linear transformation was performed on the channel descriptor through two fully connected layers so as to generate a channel weight vector with the same numbers of channels, wherein the two fully connected layers used ReLU and Sigmoid activation functions, respectively, to enhance the non-linear expressiveness and map the weight values to the [0,1] interval. Finally, the learned channel weight vector was multiplied element-wise with the original input feature map to obtain the weighted feature map, as shown in Eq. (1). By combining multi-scale feature extraction with the channel attention mechanism, the proposed module effectively captures both local and global patterns in spectral data, thereby fully exploiting its discriminative information.

(1) $$M_{c}\left(F\right)=\sigma \left(\mathrm{MLP}\left(\mathrm{AvgPool}\left(F\right)\right)+\mathrm{MLP}\left(\mathrm{MaxPool}\left(F\right)\right)\right)$$

where, $M_{c}\left(F\right)\in R^{C\times 1\times 1}$ represents the channel attention feature map, σ is the sigmoid activation function, MLP is the multilayer perceptron structure, $F\in R^{C\times H\times W}$ represents the size of the spectral feature map, and AvgPool and MaxPool are the average pooling operation and global average pooling operation, respectively.

**Table 1:** Parameters of the 1D convolutional structure.

Layer	In channels	Out channels	Kernel size	Stride	Padding	Feature size	BN?	Activation function
Conv1	1	16	7	2	1	(16,238)	yes	ReLU
Conv2	16	32	7	2	1	(32,117)	yes	ReLU
Conv3	32	64	5	2	1	(64,58)	yes	ReLU
Conv4	64	128	5	2	1	(128,28)	yes	ReLU
Conv5	128	256	3	2	1	(256,14)	yes	ReLU
Conv6	256	512	3	2	1	(512,7)	yes	ReLU
Flatten						(3584)		
FC1	3584	256				(256)	no	ReLU
FC2	256	128				(128)	no	ReLU
Share_FC	128	64				(64)	no	ReLU
Softmax	64	num_classes				(num_classes)	no	Softmax

#### 2.5.2 Image feature-extraction module

Sorghum grain images contain rich texture, shape, and color information. To fully extract these visual features, FC-RGB and IMI were selected as the input data for the 2D CNN module. To efficiently extract the image features, the lightweight CNN ShuffleNetV2 was selected as the feature extractor. ShuffleNetV2 offers the advantages of high computational efficiency and few parameters, which significantly reduce the computational overhead while ensuring feature-extraction performance (Zhang et al., 2018). Before inputting the sorghum grain images into ShuffleNetV2, a series of preprocessing operations were performed on the images, including resizing to 224 × 224, random horizontal flipping, converting to tensor format, and normalization. These operations aimed to ensure that the images had a uniform size, increase data diversity, and improve the ability of the model's generalization. In the feature-extraction process, to enhance the model's representation ability and performance, the Squeeze-and-Excitation (SE) attention mechanism was introduced. This mechanism adaptively adjusts the importance of each channel in the convolutional feature map, thereby enhancing the model's attention to key features (Hu et al., 2018). Specifically, the SE attention mechanism first performs global average pooling on the input features to obtain a global descriptor for each channel. Then, it learns the weight of each channel through two fully connected layers (dimensionality reduction and increase) and applies the Sigmoid activation function to map the weight values to the range of [0,1]. Finally, the learned channel weights were multiplied element-wise with the original input features to obtain the attention-weighted output features, as shown in Eqs. (2–5). After completing the ShuffleNetV2 feature extraction, the model passed the extracted features to a fully connected layer L2, which output 128 features. Subsequently, these features were input into a shared fully connected layer S2, ultimately outputting 64 features.

(2) $$u_{c}=v_{c}*X=\sum_{s=1}^{c^{\prime }}v_{c}^{s}*x^{s}$$

(3) $$z_{c}=F_{\mathrm{sq}}\left(u_{c}\right)=\frac{1}{H\times W}\sum_{i=1}^{H}\sum_{j=1}^{W}u_{c}\left(i,j\right)$$

(4) $$s=F_{\mathrm{ex}}\left(z,W\right)=\sigma \left(g\left(z,W\right)\right)=\sigma \left(W_{2}\delta \left(W_{1}z\right)\right)$$

(5) $${\tilde{x}}_{c}=F_{\mathrm{scale}}\left(u_{c},s_{c}\right)=s_{c}u_{c}$$

Where: * represents convolution, $v_{c}=\left[v_{c}^{1},v_{c}^{2},\cdots ,v_{c}^{C^{\prime }}\right]$, $X=\left[x^{1},x^{2},\cdots ,x^{C^{\prime }}\right]$, $u_{c}\in R^{H\times W}$, σ is the sigmoid activation function, δ is the relu activation function,$W_{1}\in {\mathbb{R}}^{\frac{C}{r}\times C}$, $W_{2}\in {\mathbb{R}}^{C\times \frac{C}{r}}$, $\tilde{X}=\left[{\tilde{x}}_{1},{\tilde{x}}_{2},\cdots ,{\tilde{x}}_{C}\right]$, $u_{c}\in R^{H\times W}$, *F*_*scale*_ is the convolution on the channel.

#### 2.5.3 Cross-modal feature fusion module

**Fig. 4:**
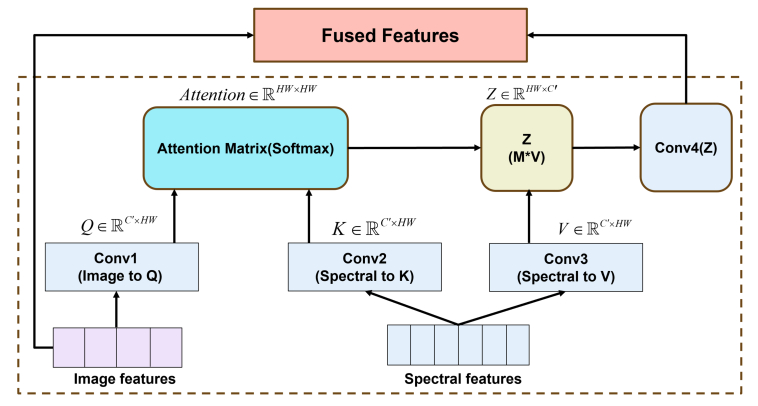
Cross-Modal Attention Fusion Module.

To effectively fuse the spectral features with the image features, a Cross-Modality Attention (CMA) fusion module was used. CMA achieves feature interaction and fusion through an attention mechanism, which adaptively extracts and fuses complementary information from different modalities (Wang et al., 2022). The structure of CMA is displayed in Fig. 4, with image features and spectral features as the inputs. In the image features processing, a convolutional layer Conv1 was used to convert the input 64-channel image feature map into a query (Q). Similarly, in the spectral features processing, two parallel convolutional layers Conv2 and Conv3 were used to convert the input 64-channel spectral feature map into a key (K) and value (V), respectively. The convolution operations are depicted in Eqs. (6–8):

(6) $$Q=\mathrm{Conv}1\left(F_{\mathrm{image}}\right)$$

(7) $$K=\mathrm{Conv}2\left(F_{\mathrm{spectral}}\right)$$

(8) $$V=\mathrm{Conv}3\left(F_{\mathrm{spectral}}\right)$$

where, F_image_ and F_spectral_ represent image features and spectral features, Conv1, Conv2, and Conv3 are the convolutional layers of kernel size 1 × 1.

By converting the image features into a query (Q) and the spectral features into a key (K) and value (V), their interactions could be effectively calculated in the attention mechanism. The attention weight matrix was calculated through matrix multiplication of the query (Q) and the key (K) and then normalized through scaling and softmax operations. The calculation formula is depicted in Eq. (9), as follows:

(9) $$\mathrm{Attention}=\mathrm{softmax}\left(\frac{QK^{T}}{\sqrt{C^{\prime }}}\right)$$

where Attention represents the attention weight matrix, $C^{\prime }$ represents the hidden channel number 64, and $\sqrt{C^{\prime }}$ represents the scaling factor.

Next, the value (V) was multiplied with the attention weight matrix A to obtain the weighted spectral feature Z. Another convolutional layer Conv4 was used to further transform the weighted spectral feature Z, and it was added to the original image feature to obtain the final fused feature F_fused._ To enhance the quality of feature representation, batch normalization (BatchNorm) and LeakyReLU activation function were applied to the fused features, as shown in Eqs. (10−11):

(10) $$Z=\mathrm{Attention}\cdot V^{T}$$

(11) $$F_{\mathrm{fused}}=F_{\mathrm{image}}+\mathrm{Conv}4\left(Z\right)$$

where V represents the value features extracted from spectral data; Attention denotes the attention weight matrix calculated from the Query of image data and the Key of spectral data; Z represents the weighted fused feature representation; Conv4(·) is a combination module of convolution and activation, used for feature transformation; F_image_ represents the features extracted from the image branch; F_fused_ is the final fused feature, obtained through residual connection with the original image features.

The CMA module can establish cross-modal attention connections between image and spectral data, enabling the model to adaptively focus on information that is more important for the current task. The input 64-dimensional image features and 64-dimensional spectral features, after query-key-value transformation and attention weighting, yielded a fused feature representation that contains complementary information from both modalities. This fusion method fully utilizes the advantages of different modal data, extracting more comprehensive and accurate features. Finally, the fused features were normalized through the softmax function to obtain the final outputs.

### 2.6 Model evaluation strategy and experimental environment

This research adopts a training strategy combining cross-validation with final model evaluation, aiming to improve the reliability and generalization ability of model performance. The dataset is randomly divided into training set (80 %) and test set (20 %) in an 8:2 ratio, with the test set serving as a completely independent evaluation dataset to ensure the objectivity of the final assessment. To enhance the stability of model evaluation, 5-fold cross-validation was implemented on the training set, striking a balance between computational efficiency and statistical reliability. The training process consists of two key stages: first, during cross-validation, each fold is trained for 200 epochs using the Adam optimizer (learning rate 0.0001), and the best model weights are saved; subsequently, the model parameters from the best-performing fold are used as initialization parameters for the final model, fully utilizing the feature extraction capabilities obtained during the cross-validation phase, and then retrained on the complete training set while monitoring the performance on the independent test set. The model structure and detailed hyperparameter configuration used in the above training process are shown in Supplementary Table 1. To more comprehensively and objectively evaluate the model's performance, accuracy, precision, and recall are adopted as the main evaluation metrics, while the F1 score was used to evaluate the performance of each category (Song et al., 2024). Classification results are visualized through confusion matrices to comprehensively assess the model's generalization ability and potential overfitting phenomena. All experiments were developed and tested in a Windows 11 64-bit operating system environment. Hardware configuration includes the 13th Gen Intel(R) Core(TM) i7-13700KF processor (3.40 GHz) and the NVIDIA GeForce RTX 4070 Ti graphics card (12GB VRAM). The experiments were based on the Python 3.9 programming language using PyCharm 2024 as the integrated development environment and then conFig.d with common Python libraries such as NumPy, Scikit-learn, and PyTorch.

## 3 Results

### 3.1 Spectral processing and feature analysis

#### 3.1.1 Spectral data processing

During the acquisition of hyperspectral images, interference from dark current and noise is inevitable, necessitating the screening of original bands to ensure data quality. Typically, at both ends of the spectral range, the imager's response is weaker, resulting in lower signal-to-noise ratios, with signals easily affected by system noise, sensor response instability, and other factors, thus lacking effective information. This study conducted quality screening of the 512 acquired bands, removing the first 9 and last 32 severely interfered bands, ultimately retaining 471 effective bands. This processing not only reduced noise interference but also enhanced the stability of spectral data and the accuracy and reliability of subsequent analysis. To verify the impact of removing the first 9 and last 32 anomalous bands on model performance, we designed a comparative experiment. The MSI-FusionNet model was trained using both the complete 512-band data and the 471-band data after removing the first 9 and last 32 bands, and evaluated on the test set. As shown in Supplementary Table 3, after removing the anomalous bands, the model's accuracy increased from 92.22 % to 93.33 %, with precision and recall also improving to varying degrees, reaching 93.92 % and 93.38 %, respectively. These results indicate that removing the anomalous bands at both ends effectively reduced noise interference and enhanced the model's stability and classification performance, verifying the necessity and effectiveness of this preprocessing step.

#### 3.1.2 Spectral feature analysis

**Fig. 5:**
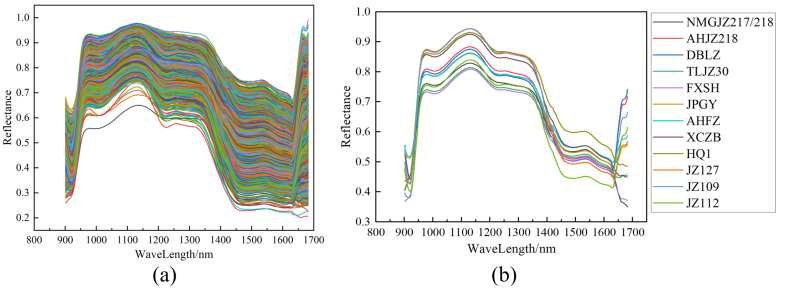
(a) Normal spectral curves of all sorghum samples. (b) average spectral curves of 12 sorghum varieties.

Fig. 5(a) presents the spectral curves of all normal sorghum samples after band filtering, while Fig. 5(b) shows the average spectral curves for the 12 sorghum varieties. Although the 12 sorghum varieties exhibit similar spectral trends, indicating common spectral characteristics—distinct variations are observed in specific spectral regions due to differences in their chemical compositions. Notable absorption peaks appear near 920, 980, 1030, 1130, 1230, and 1480 nm. The absorption peaks at 920 and 1480 nm correspond to the second and first overtone vibrations of the O—H bond, reflecting variations in moisture content (Yang et al., 2021). The 980-nm peak is associated with the second overtone stretching of the O—H bond, mainly indicating starch content (Wang et al., 2018). Absorptions near 1030 and 1130 nm result from the second overtone stretching of the C—H bond, indicating the relative proportions of carbohydrates and fats (He et al., 2021; Huang et al., 2021). The 1200–1300 nm region also reflects C—H bond activity, further indicating carbohydrate and fat presence in sorghum (Peng et al., 2024). Additionally, the 1480-nm absorption peak is related to both O—H and N—H bonds and is commonly used to assess protein content (Huang et al., 2023). Studies have similarly confirmed that the 900–1700-nm near-infrared range consistently captures these spectral patterns across sorghum samples (Fei et al., 2023; Huang et al., 2021). Within this range, spectral differences among varieties provide valuable insights into moisture, carbohydrate, fat, and protein content, offering a scientific basis for evaluating sorghum quality and varietal screening.

### 3.2 Classification modeling results of different image types based on spectral data (MSI-FusionNet)

This study introduces an MSI-FusionNet that integrates 1D spectral data with 2D image data to classify 12 sorghum varieties. The network employs multi-scale 1D and 2D convolution techniques for feature extraction. To assess the impact of image type, the study compares models using IMIs and FC-RGB images in conjunction with the same spectral data. Table 2 summarizes the performance of these models. The MSI-FusionNet model using IMIs significantly outperforms the FC-RGB-based model across accuracy, precision, and recall on both training and test sets. Specifically, the IMI-based model achieved 98.33 % accuracy on the training set and 93.33 % on the test set—5.66 % and 13.05 % higher than the FC-RGB-based model, respectively. The performance gap is primarily due to image quality. IMI images offer higher spatial resolution and clarity, capturing detailed morphological and textural features critical for differentiating sorghum varieties. By contrast, FC-RGB images lack sufficient resolution, limiting the model's ability to extract meaningful features. While hyperspectral imaging provides both spectral and image data, its 2D images are typically of lower resolution (Hu, Dai, Peng, et al., 2025). Incorporating high-resolution IMIs effectively compensates for this limitation, allowing the MSI-FusionNet model to better capture quality-related visual features.

**Table 2:** Performance of MSI-FusionNet with different image modalities (IMI vs FC-RGB) using identical spectral data.

MSI-FusionNet	Training set			Testing set		
Image type	Accuracy	Precision	Recall	Accuracy	Precision	Recall
FC-RGB	92.67 %	93.11 %	92.66 %	80.28 %	82.6 %	80.94 %
IMI	98.33 %	98.4 %	98.34 %	93.33 %	93.92 %	93.38 %

**Fig. 6:**
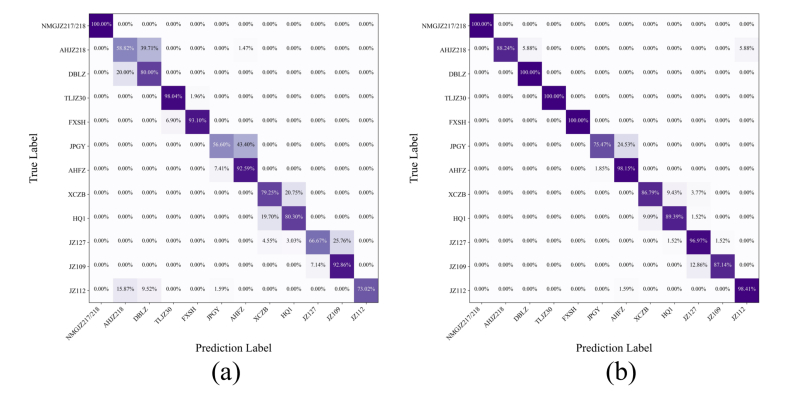
Confusion matrices of the test set. (a) FC-RGB image data, (b) IMI image data.

**Fig. 7:**
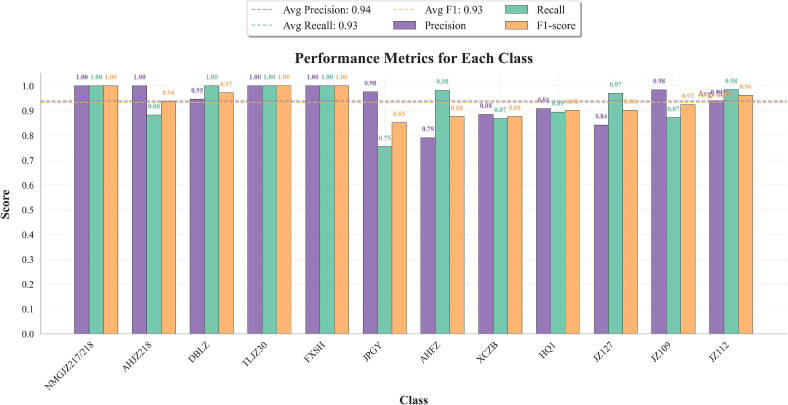
Classification performance metrics of the MSI-FusionNet model for each category.

Fig. 6 presents the confusion matrices of the MSI-FusionNet model on the test set using different image types, both combined with the same spectral data. Fig. 6a and b display the results when fusing spectral data with FC-RGB images and IMIs, respectively. Comparing these results highlights how image type influences classification performance. In Fig. 6a, while most categories are correctly classified using FC-RGB images, the model struggles with certain distinctions. Notably, approximately 39.71 % of AHJZ218 samples were confused with DBLZ, 43.4 % of JPGY samples were misclassified as AHFZ, 20.75 % of XCZB samples were mistaken as HQ1, and 25.76 % of JZ127 samples were misidentified as JZ109. These misclassifications contribute to the overall lower accuracy. By contrast, the MSI-FusionNet model using IMIs in Fig. 6b demonstrates markedly improved classification performance. All 12 sorghum categories are more accurately classified, with significantly fewer errors. The main confusions are limited to JPGY and HQ1, with 24.53 % of JPGY samples misclassified as AHFZ and 12.86 % of JZ109 samples misclassified as JZ127. Fig. 7 further compares classification performance metrics of the MSI-FusionNet model for each category—precision, recall, and F1-score. The results show high and balanced scores across most classes, indicating the model's strong classification and generalization capabilities.

In summary, fusing IMIs with spectral data substantially enhances the MSI-FusionNet model's classification accuracy, particularly for categories that are difficult to distinguish using FC-RGB images. These findings underscore the importance of selecting appropriate image types for fusion, as they provide richer and more discriminative features for hyperspectral image classification.

### 3.3 Performance comparison of MSI-FusionNet based on different 2D convolution modules

Two-dimensional CNNs have achieved remarkable success in computer vision tasks such as image classification, object detection, and semantic segmentation due to their powerful feature extraction capabilities. Building on prior work using 1D spectral data and 2D microscopic images of sorghum, the MSI-FusionNet model was developed. It achieves dual extraction and integrates 1D- and 2D-CNNs to enable simultaneous extraction and deep fusion of spectral and spatial features. To further enhance model performance, this study evaluated the impact of four representative 2DCNN modules—MobileNetV3, GhostNet, VGG16, and ShuffleNetV2—on MSI-FusionNet. Each was embedded into the 2DCNN branch of the model, while the 1DCNN component remained unchanged. Performance was evaluated on the same dataset using metrics such as accuracy, precision, recall, parameter count, model size, and average inference time (Table 3).

**Table 3:** Performance comparison of different 2D-CNN modules in MSI-FusionNet using spectral data and IMI data.

2DCNN	Accuracy	Precision	Recall	Parameter Count	Model Size	Average Inference Time (s)
MobileNetV3	88.33 %	88.50 %	88.27 %	4,811,232	18.35 MB	0.01622
GhostNet	87.92 %	88.82 %	88.18 %	7,043,216	26.87 MB	0.02278
VGG16	92.95 %	93.12 %	92.95 %	23,500,428	89.65 MB	0.01319
ShuffleNetV2	93.33 %	93.92 %	93.38 %	5,492,844	20.95 MB	0.01551

Among all models, ShuffleNetV2 achieved the highest accuracy (93.33 %), outperforming VGG16 by approximately 0.38 % and MobileNetV3 and GhostNet by approximately 5–6 %. ShuffleNetV2's superior performance can be attributed to its pointwise group convolutions and channel shuffle operations, which effectively extract spatial features without significantly increasing computational complexity. While VGG16 also delivered strong results due to its multiple 3 × 3 convolutions, it had the largest number of parameters (23.5 million) and the largest model size (89.65 MB), making it less practical for lightweight applications. By contrast, ShuffleNetV2 had the fewest parameters (approximately 5.5 million) and the smallest model size (approximately 20.95 MB), thanks to its depthwise and pointwise convolutions and channel shuffling, which can respectively reduce the number of parameters and increase information exchange between different groups, enhancing feature expression capability. GhostNet and MobileNetV3 had similar parameter counts and intermediate model sizes (approximately 7 million/27 MB and 4.8 million/18 MB, respectively), benefiting from depthwise separable convolutions, although not to the extent seen with ShuffleNetV2. Additionally, in terms of inference speed, VGG16 had the fastest average inference time (0.01319 s), followed by ShuffleNetV2 (0.01551 s), MobileNetV3 (0.01622 s) and GhostNet (0.02278 s). Despite being slightly slower than VGG16, ShuffleNetV2 offered the best balance of speed, accuracy, and model efficiency compared to MobileNetV3 and GhostNet.

**Fig. 8:**
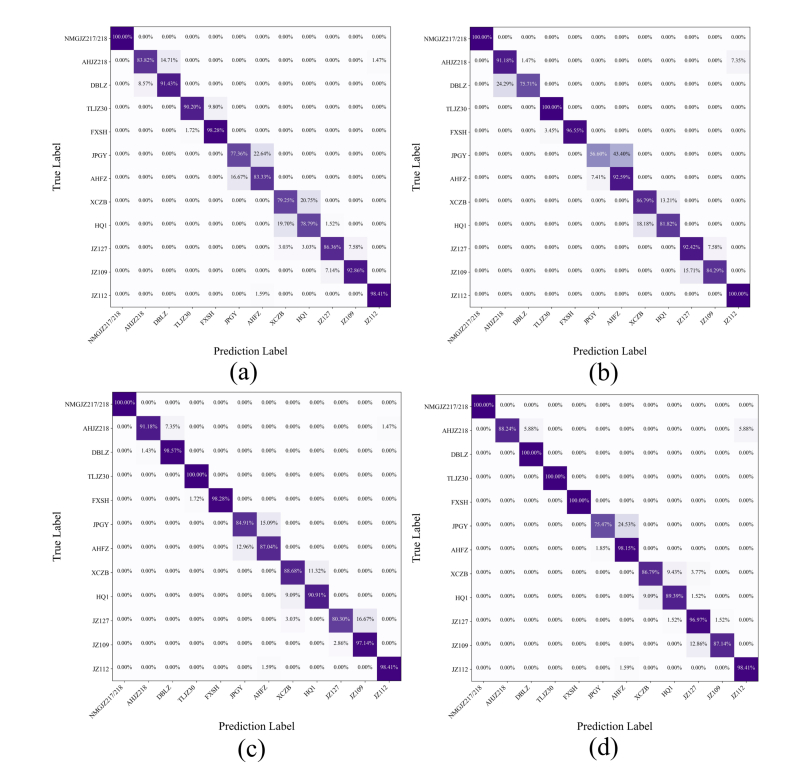
Confusion matrices of MSI-FusionNet models constructed with different 2DCNNs. (a) MobileNetV3, (b) GhostNet, (c) VGG16, (d) ShuffleNetV2.

Fig. 8 presents confusion matrices for all four MSI-FusionNet variants. Models using MobileNetV3 and GhostNet exhibited greater misclassification, particularly between JPGY and AHFZ, XCZB and HQ1, and ZJ127 and JZ109 (Fig. 8a and b). VGG16 (Fig. 8c) showed misclassification primarily between AHFZ and XCZB, and between JZ127 and JZ109. ShuffleNetV2 (Fig. 8d) mainly concentrated misclassifications between JPGY and AHFZ with approximately 24.53 % error rate.

In summary, ShuffleNetV2 emerged as the optimal 2DCNN module for MSI-FusionNet, offering superior classification accuracy, minimal parameter load, and competitive inference speed. While VGG16 also performed well, its numerous parameters and large size limit its practical use. MobileNetV3 and GhostNet offer lightweight designs but at the cost of reduced classification accuracy. These findings underscore the importance of choosing the right 2DCNN module to optimize multimodal fusion network performance.

### 3.4 Model performance comparison analysis

To comprehensively evaluate the effectiveness of multimodal spectral-image fusion for classifying 12 sorghum varieties, this study employed multi-scale 1D and 2D convolution techniques. We conducted comparative analyses of models using only 1D convolution, 2D convolution, multimodal spectral-image fusion models without attention mechanisms (MSI-FusionNet(w/o attention)), and multimodal spectral-image fusion models with attention mechanisms (MSI-FusionNet). Table 4 summarizes the classification performance across these models. The 1DCNN outperformed the 2DCNN, achieving training/testing accuracies of 86.91 %/82.22 %, precision of 87.54 %/82.69 %, and recall of 86.83 %/82.33 %. This confirms that for sorghum classification, 1D spectral data is more informative than image data (2D). However, fusing both modalities resulted in significantly improved performance. The MSI-FusionNet(w/o attention) model achieved 96.39 % accuracy on the training set and 91.39 % on the test set—improvements of 9.48 % and 9.17 % over 1DCNN, and 23.59 % and 27.69 % over 2DCNN, respectively. This highlights the effectiveness of multimodal fusion in capturing complementary features from spectral and image data. The fusion of extracted spectral features of sorghum through 1D convolution and image features through 2D convolution can effectively achieve feature complementation and enhancement, thereby improving classification accuracy. Adding attention mechanisms to both 1D- and 2D-CNNs further enhanced the model's performance. The full MSI-FusionNet achieved test set accuracy, precision, and recall of 93.33 %, 93.92 %, and 93.38 %, respectively—gains of 1.94 %, 1.84 %, and 2.11 % compared with the model without attention. These results demonstrate that attention modules help the network focus on salient features and suppress irrelevant information, enhancing overall classification performance. In conclusion, the proposed MSI-FusionNet model, particularly with attention mechanisms, achieved excellent results in the classification of sorghum varieties. Furthermore, to validate the effectiveness of the adopted fusion strategy, this study compared the performance differences between feature-level fusion and decision-level fusion using the same data combination (spectral + microscopic images) as shown in Supplementary Table S2. The results show that feature-level fusion improved accuracy, precision, and recall by 11.9 %, 11.7 %, and 11.36 %, respectively, compared to decision-level fusion. In contrast, feature-level fusion enables deep interaction of multimodal information during the feature extraction stage, more fully exploiting the complementarity between modalities, whereas decision-level fusion only integrates at the classification result level, limiting the potential advantages of modality fusion. Therefore, feature-level fusion demonstrated superior classification performance in the multimodal classification task of this study.

**Table 4:** Ablation study on the effect of attention in MSI-FusionNet and comparison with baseline CNN models.

Model	Training set			Testing set		
	Accuracy	Precision	Recall	Accuracy	Precision	Recall
1DCNN	86.91 %	87.54 %	86.83 %	82.22 %	82.69 %	82.33 %
2DCNN	72.8 %	74.04 %	72.77 %	63.7 %	64.78 %	63.74 %
MSI-FusionNet(w/o attention)	96.39 %	96.55 %	96.41 %	91.39 %	92.08 %	91.27 %
MSI-FusionNet	98.33 %	98.4 %	98.34 %	93.33 %	93.92 %	93.38 %

### 3.5 Data dimensionality reduction visualization

**Fig. 9:**
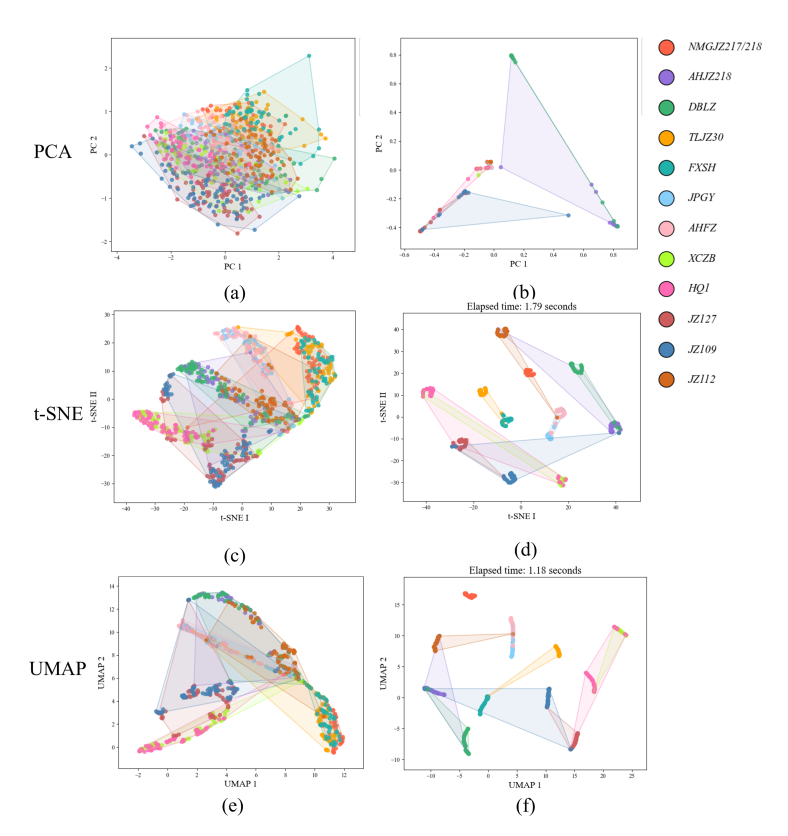
Data dimensionality reduction visualization.

To visually assess the effectiveness of the multimodal spectral-image fusion network in identifying 12 sorghum varieties, three widely used dimensionality reduction techniques were applied: PCA, t-Distributed Stochastic Neighbor Embedding (t-SNE), and Uniform Manifold Approximation and Projection (UMAP). These methods projected the data into a 2D space, allowing intuitive evaluation of the clustering and separability of features extracted by the model. PCA, a linear technique that maximizes data variance (Jolliffe, 2002), is shown in Fig. 9a and b, visualizing the original test set and the model-extracted features, respectively. In Fig. 9a, data points from different varieties are highly intermingled, showing no clear boundaries. Fig. 9b displays modest improvement with some clustering, though notable overlaps persist—highlighting PCA's limitations in capturing complex, nonlinear structures. t-SNE, a nonlinear dimensionality reduction method that preserves local structures (Van der Maaten & Hinton, 2008), shows its results in Fig. 9c and d. While Fig. 9c reveals partial clustering in the original data, Fig. 9d demonstrates significantly better separation between sorghum varieties. The model-extracted features cluster more tightly within classes and form distinct groups, indicating effective feature learning for classification. UMAP, another nonlinear dimensionality reduction method with strong global and local structure preservation (McInnes et al., 2018), is presented in Fig. 9e and f. In Fig. 9e, category boundaries remain unclear in the original data. However, Fig. 9f reveals clearer separation and more balanced inter-class distances after feature extraction, with improved structural preservation. Notably, UMAP also outperformed t-SNE in computational efficiency, requiring only 1.18 s compared to t-SNE's 1.79 s. Overall, the visualization results in Fig. 9 demonstrate that the proposed multimodal fusion network significantly enhances feature discriminability. Both t-SNE and UMAP visualizations show substantial improvements in clustering and class separation, supporting the model's robustness and effectiveness in feature extraction for sorghum variety identification.

## 4 Discussion

Multimodal fusion is a key domain in artificial intelligence that integrates data from multiple sources to achieve more comprehensive and accurate representations (Baltrušaitis et al., 2018). Deep learning plays a central role in this process by enabling robust feature extraction and representation learning across modalities, thereby improving model performance and generalization. Multimodal fusion strategies are typically categorized into early, middle, and late fusion. This study adopts middle fusion, or feature-level fusion, which preserves the individual representational strengths of each modality while enabling deeper interaction and abstraction at the feature level (Wang et al., 2025). This approach enhances model expressiveness and classification accuracy. To implement this, we developed MSI-FusionNet, which integrates 1D hyperspectral data and 2D images captured using industrial microscope cameras. Feature extraction is performed using both 1D and 2D-CNNs, followed by fusion and classification. Experimental results showed that MSI-FusionNet achieved 93.33 % accuracy in identifying 12 sorghum varieties. This represents an improvement of 11.11 % and 29.63 % over using spectral or image data alone, respectively—demonstrating the clear advantage of multimodal fusion.

Recent research on sorghum variety classification has also verified the potential of multimodal methods. For instance, Huang et al. combined spectral data and texture features through multimodal data fusion to achieve sorghum variety classification with an average accuracy exceeding 91 % (Huang et al., 2022). Zhao et al. integrated hyperspectral data and image data to develop a deep learning framework for identifying six sorghum varieties, achieving a classification accuracy of 98.64 % (Zhao et al., 2024). These studies further confirm that multimodal fusion, by integrating multi-source information, significantly enhances the performance and robustness of sorghum variety classification. Notably, compared to Zhao et al.'s study, the classification task in this study, involving 12 sorghum varieties, is more challenging, demonstrating that the proposed MSI-FusionNet exhibits strong generalization capability in handling complex classification problems. These findings align with previous research in other domains such as corn variety classification (Ge et al., 2021; Li et al., 2025), fish species recognition (Huang et al., 2025), and medical image diagnosis (Liu et al., 2025; Narotamo et al., 2024), where multimodal fusion methods have consistently improved classification performance.

To assess the impact of different image data types on the sorghum variety recognition performance of the MSI-FusionNet model, this study compared FC-RGB images and IMIs captured using industrial microscope cameras. IMIs, captured with higher spatial resolution and richer texture, yielded significantly better results—boosting classification accuracy by 13.05 %. This supports the findings by Hu et al. (2025), who showed that high-resolution microscopic images significantly improved sorghum seed recognition (Hu, Dai, Peng, et al., 2025). We also evaluated different 2DCNN architectures with MSI-FusionNet: VGG16, MobileNetV3, GhostNet, and ShuffleNetV2. Among them, ShuffleNetV2 delivered excellent classification performance with comparable accuracy to VGG16, but with only one-fourth of the parameters and one-fifth of the model size. This greatly reduces computational cost and complexity while maintaining high accuracy, making it suitable for deployment in resource-constrained environments—an advantage consistent with previous studies (Islam et al., 2025; Jin et al., 2024). By contrast, while MobileNetV3 and GhostNet are more lightweight, they exhibited noticeably lower accuracy.

3D deep learning methods, such as 3D convolutional neural networks (3D-CNN), treat hyperspectral images as three-dimensional data cubes containing spectral and spatial information. They can simultaneously extract features across all three dimensions, achieving natural fusion of spectral and spatial information and avoiding the complexity of manually designed fusion strategies. Additionally, 3D-CNNs effectively capture local correlations within hyperspectral images and possess strong feature representation capabilities, making them suitable for joint extraction of spectral and spatial features. However, 3D-CNN models have a large number of parameters, require substantial computational resources during training, and demand a large sample size. Moreover, due to the generally limited sample size of hyperspectral datasets, complex 3D networks are prone to overfitting.

Although hyperspectral imaging technology can simultaneously acquire spectral and spatial information, its spatial resolution is relatively low, and near-infrared hyperspectral images are often pseudo-colored, failing to reflect true color information. This limitation makes it difficult to fully capture the detailed texture and morphological features of sorghum. Therefore, this study introduces high-resolution two-dimensional images acquired by industrial microscopy, combined with one-dimensional spectral data obtained from hyperspectral imaging, to construct a multimodal fusion network. This approach fully leverages the chemical composition advantages of hyperspectral spectral information and the high-resolution spatial features of industrial microscopic images, achieving accurate identification of sorghum varieties. Based on these considerations, this study employs a 1D convolutional neural network to process spectral data and a 2D convolutional neural network to process industrial microscopic images, and integrates them through cross-modal fusion to accurately identify sorghum varieties. This method not only utilizes mature 1D and 2D convolutional network techniques, reducing model complexity and computational resource requirements, but also suits scenarios with limited sample sizes, ensuring stable and efficient model training. Both previous studies and our results demonstrate that separately extracting and fusing spectral and image features can achieve high recognition accuracy with a simpler model design (Lin et al., 2025; Yin et al., 2025). Future work will explore the application potential of 3D deep learning for hyperspectral image and spectral fusion based on larger-scale hyperspectral data cubes to further improve model performance.

Although MSI-FusionNet achieving high-precision identification of baijiu sorghum varieties under controlled experimental conditions, certain limitations remain. This study's dataset only covers 12 varieties (3600 grains) and was collected solely in a laboratory environment to reduce interfering factors. However, in actual production, sorghum sources are more complex, influenced by multiple factors such as production area, harvest season, storage conditions, and environmental noise. Additionally, grain surfaces often have particle residues and quality defects, such as powder produced by insect damage, black spots, brown spots, and cracks. Although these factors in natural states may cause local spectral fluctuations, this study demonstrates that after ROI limitation, background removal, and spectral averaging processing, their impact on overall performance is limited and may even increase data diversity to some extent. In the future, more systematic quantification of the specific effects of different defects and residual particles on spectral characteristics is still needed to better evaluate the model's robustness in real environments. Future research will focus on the following aspects: first, expanding the diversity of the dataset by including varieties from more growing regions and harvest seasons, covering a broader range of species and quality differences; second, introducing multi-environment data collection to simulate different lighting, humidity, temperature, and background conditions; third, increasing multi-device data acquisition by experimenting with industrial cameras and consumer-grade RGB cameras (such as smartphone cameras), and comparing these with microscope images to verify the feasibility of low-cost data collection.

## 5 Conclusion

We here used HSI and industrial microscopic cameras to acquire 1D spectral data and 2D image data of sorghum seeds, respectively, thus constructing a multimodal data fusion model named MSI-FusionNet for identifying 12 sorghum varieties. On fusing the obtained spectral and image data, the sorghum variety identification accuracy was 93.61 %, exhibiting improvements of 11.11 % and 29.63 % over spectral-only or image-only models, respectively. Thus, the MSI-FusionNet model effectively integrates features from different model data, thereby allowing them to complement each other and significantly enhance the model's identification performance. Among four 2D-CNN backbones (VGG16, MobileNetV3, GhostNet, ShuffleNetV2**),** ShuffleNetV2 achieved the best trade-off between accuracy, parameter count (approximately 5.5 M), and model size (approximately 21 MB). It significantly reduced model complexity and computational costs while maintaining high accuracy. Data dimensionality reduction visualizations (PCA, t-SNE, UMAP) confirmed enhanced the model's feature expression capability and classification effectiveness. In the future, the performance of the sorghum variety identification model will be improved in two aspects: first, by expanding the dataset's scale and diversity, the model's generalizability and robustness can be improved; second, by optimizing the MSI-FusionNet model architecture, more efficient feature fusion strategies can be explored and the model's feature expression capability and classification accuracy can be further improved.

## CRediT authorship contribution statement

**Xinjun Hu:** Writing – review & editing, Writing – original draft. **Mingkui Dai:** Resources. **Anjun Li:** Supervision. **Ying Liang:** Supervision. **Wei Lu:** Supervision. **Jiahao Zeng:** Supervision. **Jianheng Peng:** Resources. **Jianping Tian:** Supervision. **Manjiao Chen:** Supervision. **Liangliang Xie:** Supervision.

## Declaration of competing interest

The authors declare that they have no known competing financial interests or personal relationships that could have appeared to influence the work reported in this paper.

## Data Availability

Data will be made available on request.

## Appendix A Supplementary data

Supplementary material. Image 1
