# Supplementary material for: MSI-FusionNet: a multi-modal spectral-image fusion network for sorghum variety identification
Source: Food Chem X. 2025 Oct 9;31:103137. doi: 10.1016/j.fochx.2025.103137 (PMC12546670; doi:10.1016/j.fochx.2025.103137)
Supplement: Supplementary file 1 — Supplementary material. [file mmc1.docx]

Table S1 Key Parameters Used in the MSI-FusionNet Algorithm.

| Parameter Category | Parameter Name | Value / Description |
| --- | --- | --- |
| Model Architecture | 1D convolution channels | 16 → 32 → 64 → 128 → 256 → 512 |
|  | 2D feature extractor | ShuffleNetV2 (1.5x model size) |
|  | Number of classes | 12 |
| Training Hyperparameters | Number of epochs | 200 |
|  | Learning rate | 0.0001 |
|  | Optimizer | Adam |
|  | Batch size | 16 |
|  | Loss function | CrossEntropyLoss |
|  | Cross-validation folds | 5 |

Table S2 Comparison of feature-level fusion and decision-level fusion using hyperspectral data and industrial microscope images.

| Fusion Type（Spectra + IMI） | Accuracy | Precision | Recall |
| --- | --- | --- | --- |
| Feature Fusion | 93.33% | 93.92% | 93.38% |
| Decision Fusion | 81.43% | 82.22% | 82.02% |

Table S3 Comparison of Model Classification Performance Using All 512 Spectral Bands Versus After Removing the First 9 and Last 32 Spectral Bands.

| Data Processing Method | Accuracy | Precision | Recall |
| --- | --- | --- | --- |
| All 512 Spectral Bands | 92.22% | 92.75% | 92.23% |
| After Removing Bands | 93.33% | 93.92% | 93.38% |
